# Crystal Structure of Alcohol Oxidase from *Pichia pastoris*

**DOI:** 10.1371/journal.pone.0149846

**Published:** 2016-02-23

**Authors:** Christian Koch, Piotr Neumann, Oliver Valerius, Ivo Feussner, Ralf Ficner

**Affiliations:** 1 Department of Plant Biochemistry, Albrecht-von-Haller-Institute, Georg-August-University Goettingen, Justus-von-Liebig-Weg 11, 37077, Goettingen, Germany; 2 Department of Molecular Structural Biology, Institute of Microbiology und Genetics, Georg-August-University, Justus-von-Liebig-Weg 11, 37077, Goettingen, Germany; 3 Department of Molecular Microbiology and Genetics, Institute for Microbiology und Genetics, Georg-August-University, Griesebachstr. 8, 37077, Goettingen, Germany; 4 Georg-August-University Goettingen, Goettingen Center for Molecular Biosciences (GZMB), Justus-von-Liebig-Weg 11, 37077, Goettingen, Germany; Weizmann Institute of Science, ISRAEL

## Abstract

FAD-dependent alcohol oxidases (AOX) are key enzymes of methylotrophic organisms that can utilize lower primary alcohols as sole source of carbon and energy. Here we report the crystal structure analysis of the methanol oxidase AOX1 from *Pichia pastoris*. The crystallographic phase problem was solved by means of Molecular Replacement in combination with initial structure rebuilding using Rosetta model completion and relaxation against an averaged electron density map. The subunit arrangement of the homo-octameric AOX1 differs from that of octameric vanillyl alcohol oxidase and other dimeric or tetrameric alcohol oxidases, due to the insertion of two large protruding loop regions and an additional C-terminal extension in AOX1. In comparison to other alcohol oxidases, the active site cavity of AOX1 is significantly reduced in size, which could explain the observed preference for methanol as substrate. All AOX1 subunits of the structure reported here harbor a modified flavin adenine dinucleotide, which contains an arabityl chain instead of a ribityl chain attached to the isoalloxazine ring.

## Introduction

Contrary to most eukaryotic organisms, several yeast species can utilize methanol as sole carbon and energy source, enabling such methylotrophic yeasts to occupy an ecological niche [1]. The methanol assimilation pathway is initiated by the oxidation of that alcohol to formaldehyde. This reaction is catalyzed by the FAD-dependent alcohol oxidase (AOX; EC 1.1.3.13), which belongs to the family of glucose-methanol-choline (GMC) oxidoreductases [2]. In addition to methanol, this enzyme oxidizes *in vitro* other short aliphatic alcohols such as ethanol and 1-propanol, what gave its generalized name alcohol oxidase. The AOX gene is subject to a strong carbon catabolite repression [3, 4]. Consequently its synthesis is strictly regulated by induction and repression/derepression mechanisms that occur at the transcriptional level with identified regulating components being a hexose transporter [5], a glucokinase and a hexokinase [6]. Under repression conditions, the *AOX* promoter sequence (P_AOX_) is organized in nucleosome structures and is unavailable for the cellular transcription machinery. Prior to initiation of AOX synthesis at inducing (for instance in the presence of methanol) or derepressing conditions, the gene needs to be liberated from nucleosomes via the function of the chromatin remodeling complex [7]. AOX is translated on free cytosolic ribosomes and post-translationally imported into the peroxisomal matrix. The required peroxisomal targeting signal (PTS) in *Picha Pastoris* AOX is primarily located within the four C-terminal amino acids of the protein (Leu-Ala-Arg-Phe) [8]. Interestingly, formation of functional AOX assembly can only be initiated upon targeting it into the destination organelle. Catalyzed by AOX and taking place in peroxisomes oxidation of methanol requires molecular oxygen as terminal electron acceptor, which is necessary to reoxidize the reduced flavin cofactor [8]. The concomitantly formed hydrogen peroxide is detoxified by the action of catalase, and the resulting formaldehyde may either enter a catabolic or an anabolic pathway [1]. Once induced by the presence of methanol, methylotrophic yeasts synthesize large amounts of the enzyme (up to 30% of the total soluble cellular protein [9]), putatively to compensate for relatively low affinity of AOX for oxygen [10–13]. Based on its tight regulation and strong transcriptional activation, the described system renders methylotrophic yeasts an intriguing host for biotechnological processes and especially *Pichia pastoris* is often chosen for heterologous protein expression [1, 14]. These hosts don’t only enable efficient production of post-translationally modified recombinant proteins, but also their cultivation and genetic manipulation is relatively easy as compared to other eukaryotic expression systems. Actually most of the recombinant proteins produced in these systems are expressed under the control of the methanol induced AOX1 promoter [15].

Due to their significance in biochemical processes and technical applications, alcohol oxidases from methylotrophic yeast have been extensively investigated in the past. Performed studies involved *inter alia* elucidation of their catalytic mechanism which, based on primary deuterium and solvent kinetic isotope effects, was postulated to be a hydride transfer mechanism for GMC oxidoreductases including choline oxidase [16] and AOX [17]. It was reported that enzyme’s FAD cofactor could also be modified by AOX itself [18, 19] with high FAD modification ratio at negligible methanol concentrations. This modified FAD, found so far exclusively in AOX, contains an arabityl instead of the ribityl moiety and was reported to modulate the enzyme’s activity [18]. Ultracentrifugation and electron microscopy revealed that AOX exhibits an octameric quaternary structure [10, 13, 20]. Although crystals of AOX were reported [13, 20–22], no atomic structure has been determined so far, probably due to lattice defects in the crystals [23, 24], weaker synchrotron X-ray sources without adjustable collimation, absence of large area single-photon-counting detectors as well as lack of good templates for Molecular Replacement and sophisticated model refinement strategies involving improvement of poor initial model.

Here we report expression, purification, crystallization and the crystal structure of AOX1 from *Pichia pastoris*.

## Materials and Methods

### Protein expression and purification

Expression and purification of *Pichia pastoris* AOX1 was a serendipitous result of an attempt to overexpress the heme dependent linoleate diol synthase (LDS) from *Verticillium dahlia*. Therefore *Pichia pastoris* (strain X33) was transformed with the pPICZ expression plasmid (Invitrogen; Carlsbad; USA) containing the open reading frame for VDAG_02241 (heme dependent linoleate diol synthase from *Verticillium dahlia*, Uniprot-ID: G2WVA0). Prior to transformation, this plasmid was linearized by digestion with *SacI* to facilitate insertion at the end of the AOX1 promoter. A selected recombinant colony was utilized as inoculum for the expression culture. The fermentation was performed with a 20 L BIOSTAT C Plus from Sartorius according to Invitrogen’s “Pichia Fermentation Process Guidelines”. Briefly, cells were grown in a minimal medium (26.7 mL/L H_3_PO_4_, 0.93 g/L CaSO_4_, 18.2 g/L K_2_SO_4_, 14.9 g/L MgSO_4_*7H_2_O, 4.13 g/L KOH, 26 mg/L CuSO_4_*5H_2_O, 0.35 mg/L NaI, 13 mg/L MnSO_4_*H_2_O, 0.9 mg/L Na_2_MoO_4_*2H_2_O, 0.09 mg/L H_3_BO_3_, 2.2 mg/L CoCl_2_, 87 mg/L ZnCl_2_, 283 mg/L FeSO_4_*7H_2_O, 0.9 mg/L biotin, 0.02 mL/L H_2_SO_4_), which was supplemented with either glycerol or methanol as sole carbon source. Within the first phase, biomass was generated throughout a batch-phase (6 L of minimal medium supplemented with 4% (w/v) glycerol) and a consecutive fed-batch phase (three hours of constant glycerol feed with 50% (w/v) glycerol containing named trace elements). After entire consumption of glycerol, AOX expression was induced at a bio wet weight of ~ 220 g/L by switching to methanol as feeding solution. Therefore, 100% methanol supplemented with trace elements was fed with an initial rate of 0.5 mL/min. Throughout adaptation of the cell's metabolism, methanol accumulated within the medium. Afterwards, methanol was fed growth-limiting for 35 hours. At the end of the fermentation, cells (~ 330 g bio wet weight per liter) were harvested by centrifugation and the cell pellet was stored at -80°C. Cells were disrupted mechanically by glass beads in a 50 mM phosphate buffer (pH 6.0) containing 5% glycerol (v/v). After removal of the cell debris by centrifugation (50000*g, 4°C, 20 min), the supernatant was loaded on a Source 30Q anion exchanger column (GE Healthcare, Freiburg, Germany) and elution was facilitated by a NaCl-gradient. The fraction containing the overexpressed protein was concentrated using a Spin-X UF concentrator (100 kDa cut-off; Corning Inc., USA). In the course of this concentration step protein precipitated spontaneously in the presence of NaCl and at a pH near the isoelectric point of the enzyme. The precipitate was easily resolved in 20 mM HEPES (pH 7.4) and the enzyme was further purified by size exclusion chromatography (Superdex S200 26/60, GE Healthcare, Freiburg, Germany) with 20 mM HEPES, pH7.4. The identity of the purified protein was initially assessed by estimating the molecular mass of the overexpressed protein by means of analytical size exclusion chromatography, however biochemical tests revealed no activity expected for linoleate diol synthase. Analysis by mass spectrometry revealed that the overexpressed and purified protein was *Pichia pastoris* alcohol oxidase AOX1 originating from the expression host.

### LC-MS analysis

In-gel digestion of proteins with trypsin was done according to [25]. Peptide analysis with liquid chromatography coupled to an Orbitrap Velos Pro™ mass spectrometer (Thermo Scientific) was employed for protein identification. Peptides of 2 μl sample solution were trapped and washed on an *Acclaim*^®^ *PepMap 100* pre-column (100 μm x 2 cm, C18, 3 μm, 100 Å; Thermo Scientific) at a flow rate of 25 μl/min for 6 min in 100% solvent A (98% water, 2% ACN, 0.07% TFA). Analytical peptide separation by reverse phase chromatography was performed on an *Acclaim*^®^ *PepMap RSLC* column (75 μm 15 cm, C18, 3 μm, 100 Å; Thermo Scientific) running a gradient from 98% solvent A (water, 0.1% formic acid) and 2% solvent B (80% ACN, 20% water, 0.1% formic acid) to 42% solvent B within 40 min at a flow rate of 300 nl/min. Chromatographically eluting peptides were online ionized by nano-electrospray (nESI) using the *Nanospray Flex Ion Source* (Thermo Scientific) at 2.4 kV and continuously transferred into the mass spectrometer. Full scans within the mass range of 300–1850 m/z were taken within the Orbitrap-FT analyzer at a resolution of 30,000 with parallel data-dependent top ten MS2 *collision-induced dissociation* (CID) fragmentation with the *LTQ Velos Pro* linear ion trap. LC-MS method programming and data acquisition was done with the software *XCalibur 2*.*2* (Thermo Scientific). For protein identification MS/MS2 data were searched against the Uniprot database. Mass tolerances of precursors and fragment ions were set to 10 ppm and 0.6 Da, respectively. False discovery rates were calculated with the *Proteome Discoverer* using the reverse-decoy mode, and the filter for valid peptide sequence matches was set to 0.01 FDR. The alcohol oxidase of *P*. *pastoris* (uniprot identifier P04842) was identified with a protein score of 2107, sequence coverage of 87.9%, and 553 spectral counts.

### Biochemical characterization of purified AOX1

The conversion of methanol to formaldehyde was quantitatively measured by analyzing the reaction product of the latter compound with acetylacetone in the presence of ammonium ions [26]. Dissolved oxygen was utilized as probe to continuously monitor AOX activity and the reaction was conducted in a thermostated Oxygraph from Hansatech-Instruments (Norfolk, UK) at 30°C. To measure the pH-dependency of the oxidation of 2 mM methanol solution, 67 mM phosphate-buffer was used for pH values < 8.0 and 55 mM Tris-HCl was used for pH values > 8.0. Kinetic measurements were performed in 67 mM phosphate buffer, pH 7.4. The reaction was initiated by addition of 100 nM enzyme to the buffer containing variable concentrations of substrate. Prior to the measurements, the dissolved oxygen level was calibrated by complete reduction with sodium dithionite. For evaluation a stoichiometric and equimolar redox reaction was assumed and the substrate consumption-rate was directly obtained from the measured changes of dissolved oxygen. The obtained data were fitted to a classical Michaelis-Menten equation.

### Crystallization and X-ray diffraction data collection

Prior to crystallization the enzyme was concentrated to 15 mg/ml in 20 mM Na-HEPES, pH 7.5 and supplemented with the putatively required cofactor hemin (Fluka; St. Louis; USA) at a concentration of 280 μM. Initial crystallization conditions, for suspected heme dependent linoleate diol synthase, were found by screening several commercially available crystallization kits followed by manual optimization of the best hit. Diffraction quality crystals were obtained at 4°C using sitting drop vapor diffusion setup by mixing 3μl of protein with equal amount of reservoir solution containing 100 mM HEPES, pH 7.5, 200 mM CaCl_2_ and 33.3% (w/v) PEG 400. Plate-shaped yellow crystals grew within one month to the size of 0.5 x 0.5 x 0.05 mm. Prior to data collection crystals were shortly washed in a reservoir solution supplemented with 10% (v/v) glycerol and flash-cooled by plunging into liquid nitrogen. The oscillation photographs were collected at 100 K at beamline 14.1 (BESSY, Berlin, Germany) [27]. Raw diffraction images were indexed, integrated, and scaled to a resolution of 2.35 Å using the XDS package [28, 29]. Indexing and processing of the diffraction data revealed the monoclinic space group P2_1_. Prior to use in structural refinements, 5% of the randomly selected reflections using the thin shell option were set aside for calculating R_free_ as a quality monitor [30]. The Matthews coefficient [31] suggested the presence of eight AOX1 monomers in the asymmetric unit of the monoclinic unit cell, corresponding to a solvent content of 53%.

### Structure solution and refinement

The HHPRED server [32] was used to search the PDB for closely related homologous structures and performing sequence alignment between target and template sequences. Ten best hits, PDB ids: 3FIM, 1QPE, 3QVP, 3Q9T, 2JBV, 1JU2, 1KGD, 3PL8, 1CPY, 1N4W; shared 15% to 28% sequence identity with the target sequence and covered more than 75% of it. For these a helper script from the Rosetta package [33] was applied to set up templates, used for Molecular Replacement searches, and sequence alignment files. Template preparation involved removal of unaligned residues and stripping all non-identical side chains to the gamma atom. Due to high number of molecules occupying the asymmetric unit, MR searches using PHASER [34] were restricted to locate up to three monomers. The best MR solution originated from template derived based on structure of formate oxidase (PDB id: 3Q9T), which shares 23% of identical residues with AOX1 (545 aligned residues), but is significantly smaller than AOX1. It was identified based on LLG, TFZ score and smallest number of Cα-Cα clashes between molecules and their symmetry mates when comparing individual searches performed for all templates. In detail, the search yielded seven partial solutions with similar LLGs and TFZ scores ranging between 5.5 and 7.1 for individual molecules. Each of those solutions, comprising three positioned template molecules, had identically placed the first AOX1 molecule, thus when displayed together they built up one complete tetramer and ¾ of the second one. The missing fourth monomer of the incomplete tetramer couldn’t be properly localized using PHASER, most probably due to higher level of positional disorder in the crystal lattice or larger number of Cα-Cα clashes between molecules. Therefore, the missing AOX1 monomer was manually placed based on a superposition between incomplete and complete AOX1 tetramers. The eight AOX1 monomers occupying the asymmetric unit form a compactly packed octamer exhibiting 42 molecular symmetry (two tetramers positioned face to face to each other). Extensive refinement of an initial model using PHENIX [35] at 2.5 Å resolution resulted in poor electron density maps (mean FOM of 0.21) and high R and R_free_ factors of 50.02% and 51.96%, respectively. To overcome the difficulties in manual model rebuilding, one AOX1 monomer was subjected to energy and density-guided refinement in Rosetta utilizing constrains from averaged electron density maps calculated with CNS program [36]. Based on their Rosetta score, five hundred highest ranked models were used to generate AOX1 homo-octamers, which were then subjected to combined rigid body and grouped B-factor refinement using PHENIX. A single AOX1 monomer of the refined octameric model with the lowest R and R_free_ factors (41.72% and 43.57%, respectively, mean FOM of 0.49) was subsequently subjected to the second round of modeling using Rosetta. Employed iterative rebuild and refine protocol was constrained by newly calculated averaged electron density map. During this step the Rosetta loop modeling protocol [37] combined with cyclic coordinate descent closure [38] was used to rebuild regions around insertions and up to eight amino acid long gaps in the initial sequence alignment. The best AOX1 monomer improved by Rosetta was identified in the same way as after the first density-guided refinement and was subjected to extensive reciprocal space refinement using PHENIX. This resulted in a great improvement of electron density map quality (mean FOM of 0.61) and reduction of the R and R_free_ factors to 37.09% and 38.79%, respectively. The model was manually completed and verified against simulated annealing (SA) omit maps in Coot [39] and further refined with PHENIX. Non-crystallographic symmetry restrains were released for about 100 side chains exhibiting different conformations in eight AOX1 monomers. Initially planar restraints of the entire isoalloxazine ring of FAD were used, however mFo-DFc difference electron density map contoured at 3σ clearly revealed significant bending along the N5-N10 axis as is often observed in reduced flavoproteins [40]. The manual fit of the isoalloxazine ring required removal of the planarity restraints from the pyrimidine ring while keeping planar restraints for the dimethylbenzene and piperazine rings. The final model consisting of residues 2 to 663 for each monomer, 8 FAS molecules (modified FAD molecule with an arabityl chain attached to the isoalloxazine ring), 18 PEG molecules of different length, 2 glycerol molecules, two phosphate ions, 1 chlorine ion, 8 calcium ions and 2084 solvent atoms was refined at 2.35 Å resolution to crystallographic R and R_free_ values of 17.74% and 20.49%, respectively. Details are presented in Table 1. PISA software was used to analyze macromolecular interfaces [41]. Figures were prepared using Pymol (http://www.pymol.org). Atomic coordinates and structure factors were deposited in the Protein Data Bank under PDB id code 5HSA.

**Table 1:** Data collection and refinement statistics.

Wavelength (Å)	0.9184
Resolution range (Å)	40.88–2.35 (2.45–2.35)
Space group	P 21
Unit cell (Å, °)	117.10 165.19 164.31 90.00 95.668 90.00
Unique reflections	254643 (29314)
Multiplicity	3.2 (2.8)
Completeness	0.99 (0.97)
Mean I/sigma(I)	9.8 (1.7)
Wilson B-factor (Å^2^)	34.52
R-merge (%)	10.7 (78.2)
R-meas	12.8 (96.5)
CC_1/2_	99.4 (51.1)
Reflections used in refinement	254636 (24927)
Reflections used for R-free	12276 (1247)
R-work	0.1774 (0.2999)
R-free	0.2049 (0.3290)
CC(work)	0.965 (0.702)
CC(free)	0.956 (0.644)
Number of non-hydrogen atoms	44407
macromolecules	41607
ligands	716
Protein residues	5296
RMS (bonds) (Å)	0.005
RMS (angles) (°)	0.90
Ramachandran favored (%)	97.0
Ramachandran allowed (%)	3.0
Ramachandran outliers (%)	0
Rotamer outliers (%)	3.5
Clashscore	3.94
Average B-factor (Å^2^)	44.33
macromolecules	44.55
ligands	47.28
solvent	38.80
Number of TLS groups	64

Statistics for the highest-resolution shell are shown in parentheses.

## Results and Discussion

### Biochemical characterization of *P*. *pastoris* AOX1

An enzymatic activity assay with purified *P*. *pastoris* AOX1 (as described in section Biochemical characterization of purified AOX1) revealed that the enzyme is active at pH 7.4 and 30°C exhibiting a K_M_ value of 0.6 mM and a k_cat_ of 343 min^-1^ for the substrate methanol under these conditions (S1 Table). These kinetic parameters are in reasonable agreement with previously reported values (Zhang *et al*. reported a K_M_ of 0.6–1.0 mM and a k_cat_ of 136–270 min^-1^ for conversion of methanol at 30°C and pH 7.5) [24]. Conversions of ethanol, 1-propanol, 1-butanol and 1-pentanol demonstrated that the oxidation of different primary alcohols is catalyzed with an inverse correlation between chain length and catalytic efficiency expressed as k_cat_/K_M_. Interestingly, the decreased catalytic efficiency originates almost exclusively from a lower value of K_M_ for the longer substrates (S1 Table). 1,6-hexanediol and also isobutanol, the simplest primary alcohol with a branched alkyl chain, were oxidized, however, their oxidation rate was too low to be quantified. Notably, neither oxidation of secondary alcohols nor conversion of glycerol was observed, which might indicate that substrate binding is obstructed, if C_α_ is functionalized with a hydrophilic moiety or larger branched groups causing steric hindrance.

### Structure determination

Anisotropically diffracting, plate-shaped crystals of AOX1 from *P*. *pastoris* were obtained in a buffer containing CaCl_2_ and PEG400. These crystals usually consisted of a stack of several parallel thin layers limiting crystal’s quality. As a consequence, diffraction images frequently showed multiple lattices, split spots and high crystal mosaicity. Therefore diffraction data collection strategy required choosing proper crystal to detector distance combined with oscillation range reducing the number of spatial overlaps of recorded reflections. Of importance was using a beamline equipped with a single-photon-counting detector with large active area (PILATUS 6M) and collimated (30–100 μm diameter) beam size, which allowed data collection from different spots of the crystal [27]. Initially a tetragonal symmetry was used for processing diffraction data, however, after careful inspection of several data sets measured from different crystals, the monoclinic space group P2_1_ with unit cell dimensions a = 117.1 Å, b = 165.19 Å, c = 164.31 Å, β = 95.67° turned out to be the correct one. This crystal form is clearly different from the AOX crystals previously reported, which belonged either to space group P2_1_, but with a unit cell dimensions of a = 157.3 Å, b = 171.45 Å, c = 231.6 Å, β = 94° [21], or to space group P4_1_2_1_2 or P4_3_2_1_2 with unit cell parameters a = b = 228 Å, c = 456 Å [22]. The crystallographic phase problem was solved by Molecular Replacement using as search model the structure of a formate oxidase monomer (PDB id: 3Q9T; for details see Materials and Methods section Structure solution and refinement). The final model of AOX1 was refined at a resolution of 2.35 Å to R and R_free_ factors of 0.1774 and 0.2049, respectively. Details on X-ray data collection and refinement statistics are summarized in Table 1.

### Structure of homo-octameric AOX1

**Fig 1:**
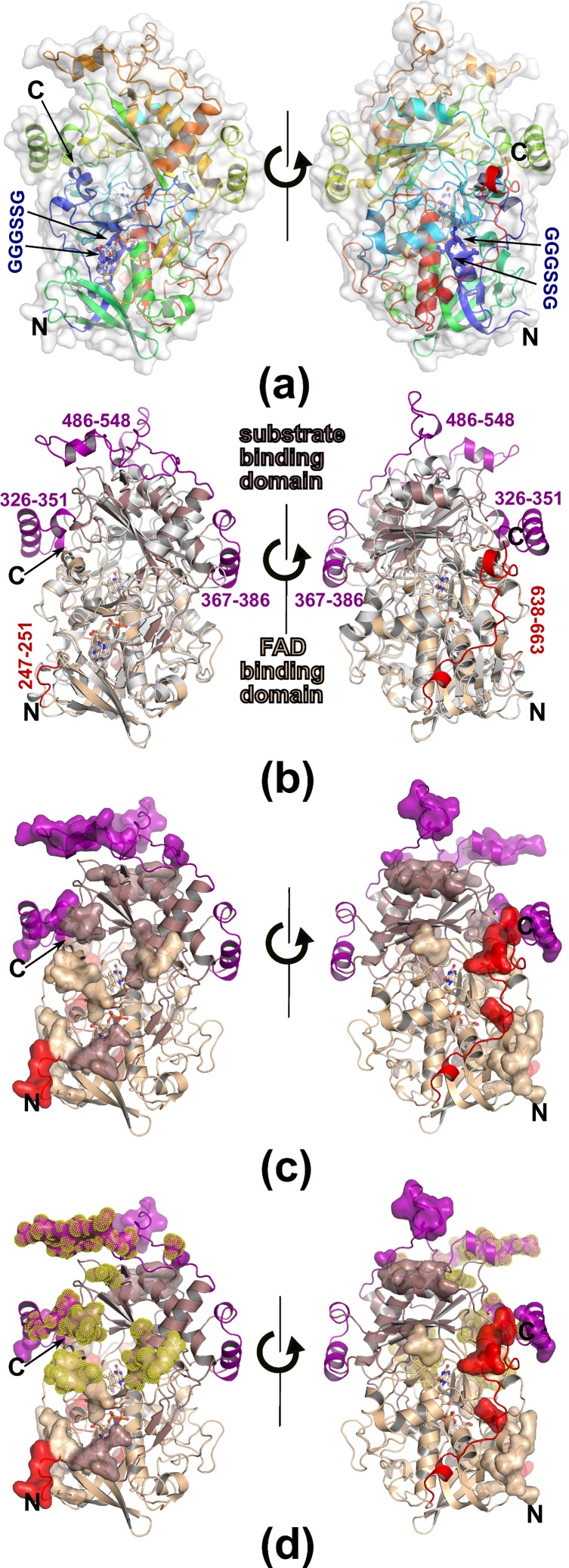
The structure of one *P*. *pastoris* AOX1 subunit. The two views are related by rotating the molecule by 180 degrees. (a) Rainbow colored cartoon representation of the AOX1 monomer. FAD is depicted in ball-and-stick representation; the position of the PP loop (residues 13–17) is highlighted as blue balls and sticks. N- and C-termini are labeled. (b) Superposition with Choline oxidase (PDB: 3LJP) and domain organization of the AOX1 subunit, both proteins represented as cartoon. The FAD-binding domain (AOX1, residues 1–155, 192–306 and 568–663) is colored wheat and a substrate-binding domain (AOX1, residues 156–191 and 307–567) is colored dirty violet. Residues forming insertions are colored purple and red for substrate- and FAD-binding domains, respectively. Choline oxidase is colored grey. (c) Monomer of AOX1 with residues involved in octamer formation depicted as surface representation, colored as in b (d) Monomer of AOX1 with residues involved in dimer formation (inter tetramer interactions) highlighted with yellow dots. Colors are chosen as in (b) and (c).

**Fig 2:**
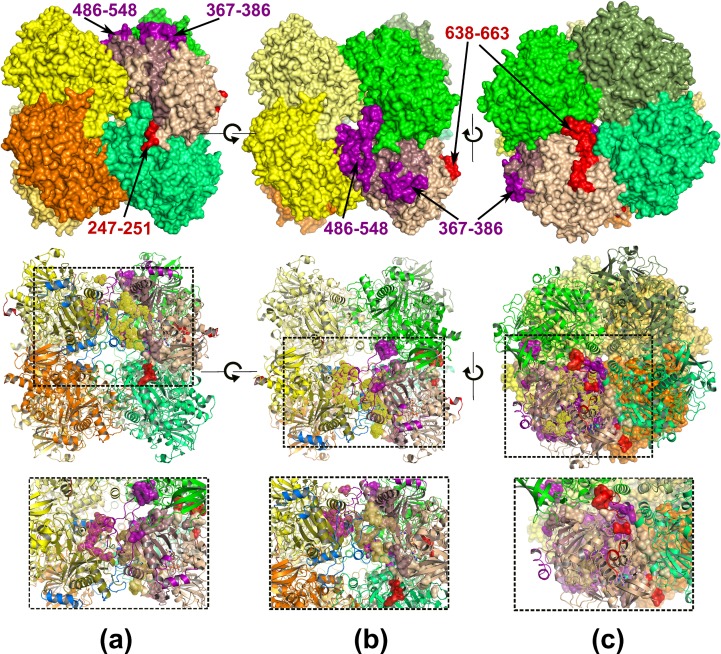
The quaternary structure of *P*. *pastoris* AOX1. Top view represents an octamer with individually colored monomers in surface representation, of which one monomer is colored as in Fig 1C. The middle view represents the cartoon representation of the octamer with one inter-tetramer dimer highlighted by different coloring: one monomer as in Fig 1D and the second one with substrate binding domain colored in dark olive and insertions in marine. Zoomed in view of the marked rectangular area depicting more detailed intersubunit interactions is presented beneath the individual octamer orientation. (a) View along 2-fold axis (b) AOX1 octamer upon rotation the “a” view by 90 degrees. (c) AOX1 octamer viewed along the 4-fold axis upon rotation the “b” view by 90 degrees. The second tetramer is depicted as surface.

Like other members of the glucose-methanol-choline (GMC) oxidoreductase superfamily, AOX1 exhibits the characteristic two-domains topology of the PHBH fold [42], containing a FAD-binding domain (residues 1–155, 192–306 and 568–663) and a substrate-binding domain (residues 156–191 and 307–567) (Fig 1A and 1B). The most conserved region is the FAD binding domain comprising four sequence fragments distributed over the whole primary sequence with the characteristic FAD nucleotide-binding site sequence (GXGXXG) of GGGSSG in *P*. *pastoris* AOX1 (Fig 1A, depicted as blue colored ball-and-stick). A structural similarity search was performed using the DALI server [43] and revealed the homo-dimeric choline oxidase (CO) (PDB-id: 3LJP, 3NNE) [44, 45], with a Z-score of 53, to be the closest structural homolog, indicated by the root-mean-square deviation (RMSD) of 1.9 Å for 500 common C_α_ atoms, even though AOX1 (663 residues) is significantly larger than CO (530 residues). Most of the additional 133 residues form four insertions and a C-terminal extension (residues 638–663) (Fig 1B). The three major insertions correspond to residues 326–351 (with a small helix 326–330 and a long helix 333–345), 367–386, and 486–548 constituting the substrate-binding domain, while only one short loop elongation (residues 247–251) is a part of the FAD binding domain (Fig 1B–1D). The C-terminal extension containing the peroxisomal targeting signal (PTS) [8] is involved in octamer formation (Figs 1D and 2C). Interestingly upon enzyme oligomerization the PTS (residues 660–663) gets buried inside the octamer. This could explain formation of functional AOX assembly only upon targeting the protein into peroxisomes.

The mature active form of AOX1 is an oligomer comprising eight identical subunits, each carrying one non-covalently bound flavin adenine dinucleotide (FAD) molecule as prosthetic group. Analysis of the crystal packing revealed that *P*. *pastoris* AOX1 crystallized as a homo-octamer with approximate dimensions of 121.8 Å x 133.8 Å x 134.5 Å (Fig 2), which is consistent with previous studies on the AOX oligomerization based on ultracentrifugation and electron microscopy [10, 13, 20]. The octamer appears to be a tetramer of dimers, but can also be described as two tetramers positioned face to face to each other. The two tetramers interact exclusively via the substrate binding domains, while the FAD binding domains occupy the outer surface of the oligomer (Fig 2). Within a tetramer, every monomer forms 20 hydrogen bonds and 8 salt bridges to each of two neighboring subunits and buries 7% (1985 Å^2^) of its accessible surface area in a pairwise interaction. In contrast, each inter-tetramer dimer is stabilized by more extensive inter-subunit contacts (40 hydrogen bonds), as also indicated by the burial of 10% (2700 Å^2^) of the accessible surface area. Upon octamer formation, each monomer forms about 115 hydrogen bonds and 16 salt bridges with neighboring subunits and buries about 29% (8300 Å^2^) of its accessible surface area indicating the strengths of the octameric assembly. Comparison with the structure of CO, the most closely related structural homolog, indicates that formation of the observed oligomerization state of AOX1 is mainly facilitated by insertions involved in numerous interactions between monomers (residues 326–351, 486–548, 247–251, 638–663) (Figs 1B–1D and 2). While one insertion located in the substrate-binding domain (367–386) forms a helix-loop-helix motif positioned distant from other monomers and thus not involved in any inter-subunit interactions, the remaining two inserts (326–351, 486–548, so-called enabling loops) are involved in formation of both tetrameric and dimeric sub-assemblies of AOX1 octamer (Fig 2). Combination of their length and built in structural plasticity with the face-to-face orientation of monomers facilitates creation of an extensive network of mutual interactions, where each insert is being involved in stabilization of the two sub-assemblies simultaneously (Figs 1D and 2). In contrast, distantly positioned short loop elongation (residues 247–251) and the C-terminal extension of the FAD binding domain are exclusively involved in the formation of the tetrameric sub-assembly by participating in interactions with two adjacent monomers forming the tetramer.

### Modified FAD cofactor

**Fig 3:**
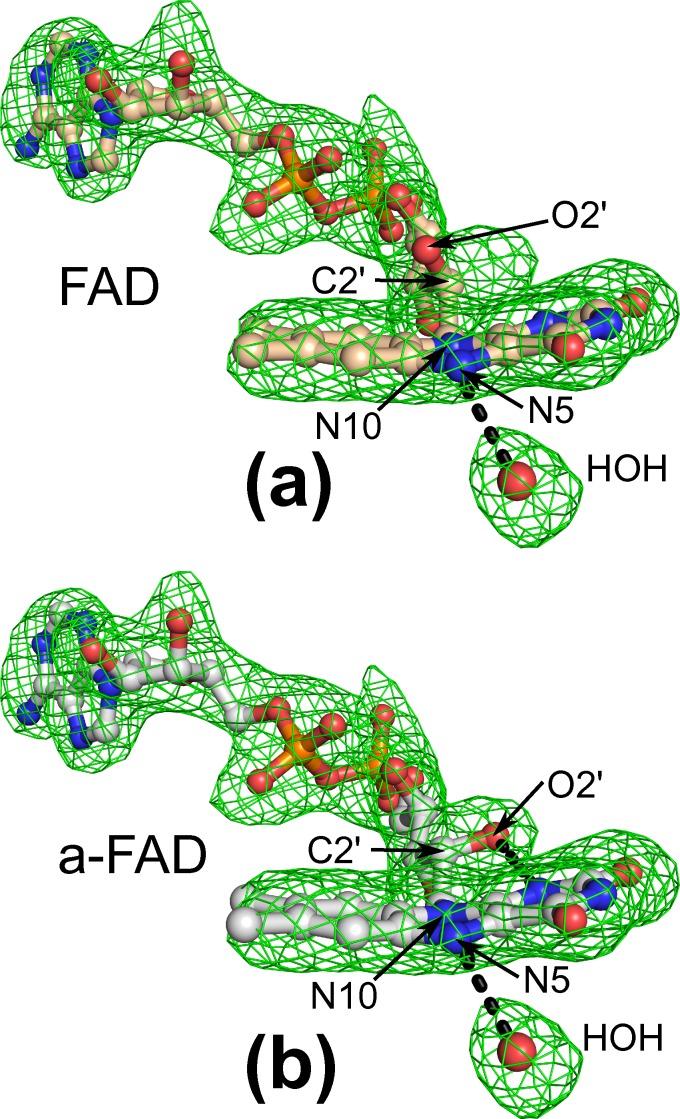
Modified a-FAD. Calculated mFo-DFc omit electron density map contoured at 3.5 σ level clearly indicates the presence of modified a-FAD molecule bound to the active site of AOX1 monomer. The bound cofactor molecule is presented in ball-and-stick representation and the accompanying water molecule is depicted as a red sphere. Atoms of the isoalloxazine ring that are important for catalysis are marked (N10, N5) as well as carbon (C2’) and oxygen (O2’) atoms affected by epimerization. (a) Not modified FAD cofactor. (b) Modified a-FAD, the changed chiral configuration center is labeled as C2’.

All eight AOX1 subunits of the reported structure contain a non-covalently bound FAD molecule in their catalytic center. Interestingly, the omit mFo-DFc difference electron density map contoured at 3.5 sigma level clearly indicates presence of a modified FAD (Fig 3), in which the configuration of the C2’ carbon of the sugar chain attached to the isoalloxazine ring is changed from *R* to *S*, hence being an arabityl rather than a ribityl chain. The absence of any positive and negative mFo-DFc electron density peaks at 3/-3 sigma levels close to C2’ carbon of arabinoflavin (a-FAD) molecules in all eight AOX1 subunits indicates high occupancy of the modified FAD present in the crystallized protein. The stereochemistry of this modified FAD in AOX was previously elucidated [46], and the amount of a-FAD in AOX, which may vary from 5% to 95%, was reported to be inversely correlated with the methanol concentration in the growth medium [18, 19]. Consequently, as the enzyme used for crystallization was derived from a methanol-limiting fermentation regime, the crystallized enzyme most likely possesses up to 95% of an a-FAD bound in the active site. Interestingly, the conversion of natural FAD into a-FAD is autocatalyzed by AOX [18, 19]. However, the mechanism of this epimerization has remained elusive. Inspection of the FAD binding pocket and comparison with the closely related CO reveals that in AOX1 the binding pocket surrounding the ribityl chain is less narrow than in CO, as Cys96 in CO is a glycine (Gly93) in AOX1, and Arg89 in CO is an alanine in AOX1 (Ala87). The side chain of Arg89 is located opposite to C2’ and C3’ atoms, while in AOX1 a large cavity is occupied by several water molecules.

### Active site and catalytic mechanism

**Fig 4:**
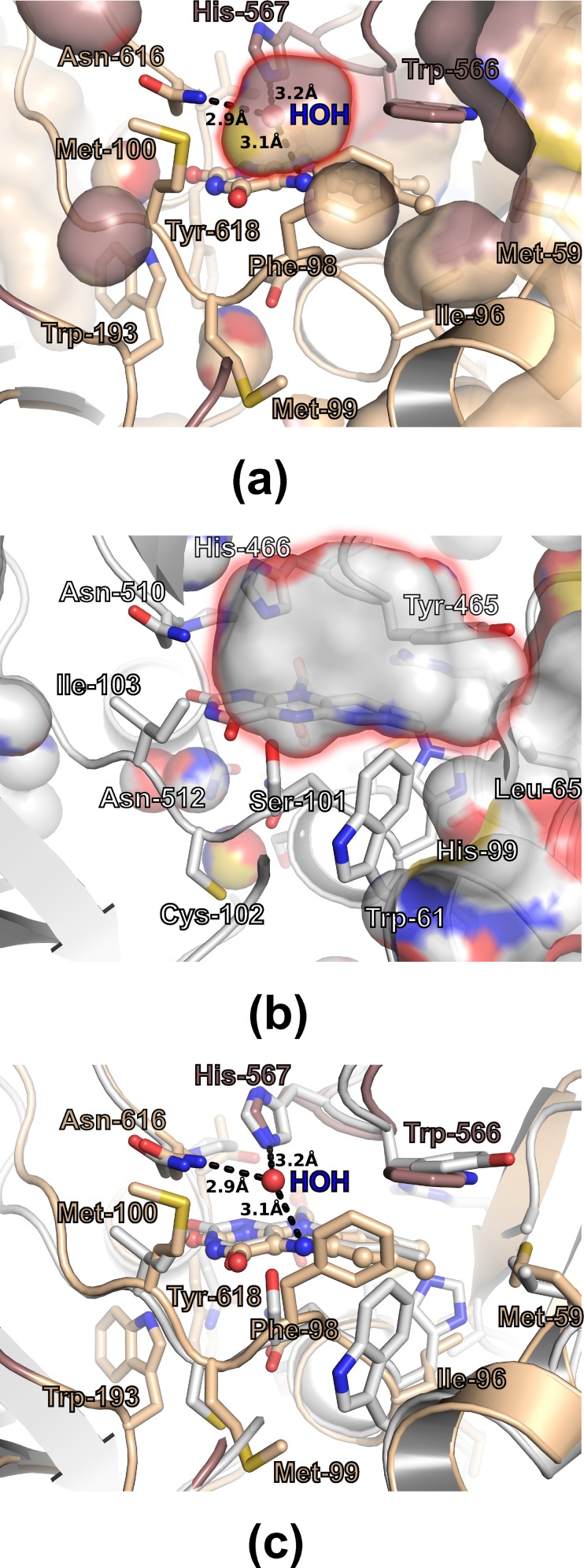
Comparison of AOX1 and choline oxidase active sites. AOX1 monomer is colored as in Fig 2C, the CO is colored grey. The residues delimiting the substrate binding cavity are labeled and represented as sticks. Modified a-FAD is depicted in ball-and-stick representation. (a) The active site of AOX1 with cavities depicted in atom colored surface representation. The substrate binding cavity is marked with blurred red rim. The water molecule, which is bound close to the isoalloxazine ring, is depicted as sphere. Polar interactions are marked as dashed lines. (b) The active site of Choline oxidase with cavities depicted in surface representation. The substrate binding cavity is marked with blurred red rim. (c) Cartoon representation of superimposed AOX1 and Choline Oxidase monomers.

**Fig 5:**
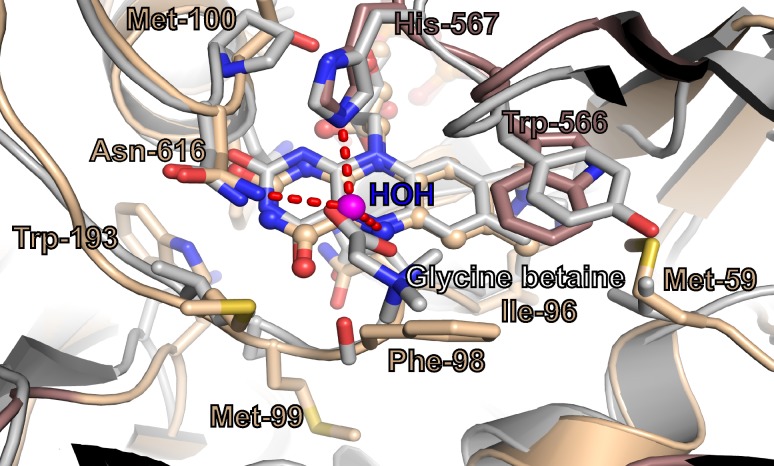
Active site comparison between AOX1 and choline oxidase complexed with glycine betaine. AOX1 monomer is colored as in Fig 2C and Fig 4, the CO is colored grey. Residues delimiting the substrate binding cavity are labeled and represented as sticks. Modified a-FAD is depicted in ball-and-stick representation. Superposition with the closely related CO in a complex with product glycine betaine (PDB id: 4MJW) indicates that the water molecule in the AOX1 active site, depicted as magenta sphere, occupies similar position as one oxygen of the carboxylic group of the bound product molecule.

Analysis of the crystal structure of AOX1 reveals that the active site is completely solvent inaccessible (Fig 4A). This interesting feature, reported also for the octameric vanillyl alcohol oxidase (VAO) [47], seems to be not related to the higher oligomerization state, as the FAD molecule in monomeric and dimeric oxidases has been observed to be either secluded from the bulk solvent in its closed configuration or exposed to solvent in its open conformation, e.g. in CO (Fig 4B). Although the AOX1 catalytic center is solvent inaccessible and delimited mainly by hydrophobic and aromatic residues, in all active sites an electron density peak corresponding to a water molecule could be observed (Figs 3 and 4A). This water molecule occupies a small cavity having a volume of approximately 135 Å^3^ (Fig 4) and it is hydrogen bonded to side chains of Asn616, His567 and to N5 atom of the isoalloxasine ring (Fig 4A). Superposition with the structure of the closely related CO in a complex with glycine betaine (PDB id: 4MJW [48]) (Fig 5) revealed, that the water molecule in the AOX1 active site occupies similar position as one oxygen of the carboxylic group of the bound product molecule in CO. This suggests that the bound water molecule could mimic the position of the formaldehyde oxygen in AOX1. Importantly, the substrate-binding pocket of AOX1 is reduced in size with regard to CO, due to the replacement of CO Ser101 to AOX1 Phe98, CO Tyr465 to AOX1 Trp 566, and CO Thr 376 to AOX1 Phe417, and in addition is more hydrophobic what could explain the preference for methanol as substrate (Fig 4C). Further inspection of the AOX1 structure suggests side chains of Phe98 and Met100 as possible structural elements whose conformational dynamics may allow substrate admission into the active site (Fig 4A and 4C).

Methylotrophic yeasts evolved a regulatory mechanism of fine-tuning the alcohol oxidase enzyme activity via autoconversion of the naturally occurring ribityl FAD moiety to the modified arabityl FAD (a-FAD). The a-FAD slightly decreases the V_max_ and significantly lowers the K_m_ value of the enzyme for the substrate methanol, which was proposed to be of physiological relevance in cultures of low methanol concentration [18]. As the multistep reaction catalyzed by AOX directly depends on the redox potential of the bound cofactor, an increase of the FAD redox potential will contribute to the overall turnover of the enzyme. Importantly, the change in chiral configuration at atom C2’ in AOX’s FAD results in the formation of a new intramolecular hydrogen bond between the arabityl C2’ OH group of the modified a-FAD and the N1 atom of the isoalloxazine ring system (Fig 3B), which most likely modulates the redox potential of the FAD cofactor. A correlation between FAD redox potential and enzyme activity is well known for other oxidases, e.g. the covalent flavinylation in CO is crucial for effective catalysis as it boosts the oxidative power of the enzyme by increasing the FAD redox potential [49, 50]. Covalent linkage of FAD is only one of the possible strategies evolved by enzymes. The flavin reactivity can also be modulated by interactions between the redox-active isoalloxazine ring and protein atoms, e.g. an increase in the redox potential was observed for any interaction lowering the electron deficiency of the pyrimidine ring [51]. In *E*. *coli* pyruvate oxidase (POX) the FAD C4’ OH group forms an intramolecular hydrogen bond with the N1 atom of isoalloxazine ring [52]. This hydrogen bond strictly depends on the conformation of the ribityl chain, which is dictated by the shape of the FAD binding site in POX, and it remains fixed once the cofactor is bound and thus, causes a permanent increase in reactivity. Interestingly, the modified arabityl has exclusively been found in AOX raising the question on the prerequisites for this auto-epimerization reaction. This leads to the speculation that the catalytic mechanism of AOX1 could indeed be slightly different than that of other alcohol oxidases. However, due to versatility of flavin reactivity and current lack of detailed kinetic analysis providing unequivocal evidence, it is difficult to reach final mechanistic conclusions about the oxidation of methanol by AOX1 and regulation of that process. Based on a primary isotope effect, a hydride transfer mechanism was suggested for GMC oxidoreductases including the related CO [16] and also for AOX [17]. According to the proposed mechanism the catalyzed oxidation reaction starts with removal of the substrate hydroxyl proton by the catalytic base and formation of an alkoxide species before the hydride transfer from the substrate α-carbon takes place. For other alcohol oxidases (AAO, GO, CDH, P2O, PNO) the conserved His of the His/His, His/Asn or His/Pro pairs is thought to act as catalytic base and thus carrying a partial positive charge during catalysis. Recently His466 of the closely related CO was identified as the catalytic base for the activation of the alcohol [53], which corresponds to His567 in AOX1 (Fig 4C). Suggested stepwise reaction catalyzed by AOX1 requires stabilization of the negative charge of the alkoxide by the enzyme through intermolecular interactions, which could be formed by two conserved polar residues, namely His567 (CO His466) and Asn616 (CO Asn 510), placed within a hydrogen bond distance to the proposed alkoxide position. As the hydride transfer appears to take place via the N5 position of the isoalloxazine ring, a polar group in close vicinity to N5 atom is expected to stabilize the transition state. In CO a properly positioned OH group belongs to Ser101, however, the corresponding residue in AOX1 is Phe98. Kinetic studies on the S101A variant of CO revealed a significant decrease in the overall rate of turnover accompanied with increased efficiencies in the oxidative half-reactions and decreased efficiencies in the reductive half-reactions [45]. Those experiments have disclosed the importance of the protein polar group close to C4a and N5 atoms of the isoalloxazine ring for the fine-tuning and optimization of the overall turnover of CO. Thus, the observed structural difference (AOX Phe98 versus CO Ser101) does not exclude a stepwise hydride transfer reaction in AOX1. However, it might explain the relative low activity of AOX in comparison to other oxidases. Nevertheless, details regarding the catalytic mechanism of AOX have to be clarified by future experiments.

## Conclusion

Taken together, the crystal structure of the homo-octameric AOX1 reveals new subunit organization, which has not been observed for other members of the GMC oxidoreductase family so far. Facilitated by insertions and a C-terminal extension octamer formation induces additional structural changes, which were not expected considering the level of sequence conservation between the target structure and potential templates used for Molecular Replacement searches. Thus, in addition to commonly used MR results validation strategies, identification of the best initial solution could additionally be facilitated by combining refinement and real space rebuilding employing energy and density-guided refinement in Rosetta program.

By these means, a crystal structure of *P*. *pastoris* AOX1 could be solved for the first time. The obtained structure might pave the way to a better understanding of the molecular mechanisms of a key enzyme of methylotrophic yeasts.

## Accession number

Coordinates and structure factors have been deposited in the Protein Data Bank with the accession code 5HSA.

## Supporting Information

S1 Table Kinetic parameters for the conversion of various substrates by *P*.*pastoris* AOX1. (DOC)

## Abbreviations

AOX: alcohol oxidase
AAO: aryl-alcohol oxidase
CO: choline oxidase
FAD: flavin adenine dinucleotide
GMC: glucose-methanol-choline
GOD: glucose oxidase
LOD: limit of detection
LOQ: limit of quantification
MR: molecular replacement
PDB: Protein Data Bank
POX: pyruvate oxidase
VAO: vanillyl alcohol oxidase
