# Supplementary material for: Crystal Structure of Alcohol Oxidase from Pichia pastoris
Source: PLoS One. 2016 Feb 23;11(2):e0149846. doi: 10.1371/journal.pone.0149846 (PMC4764120; doi:10.1371/journal.pone.0149846)
Supplement: S1 Table — (DOC) [file pone.0149846.s001.doc]

# Supplemental Material

**Table S1: Kinetic parameters for the conversion of various substrates by *P.pastoris* AOX1.**

| Substrate | k_m_ | k_cat_ | k_cat_/k_m_ |
| --- | --- | --- | --- |
| Methanol | 0.6 mM | 343 min^-1^ | 575 mM^-1^* min^-1^ |
| Ethanol | 7.9 mM | 331 min^-1^ | 42 mM^-1^* min^-1^ |
| 1-Propanol | 21.7 mM | 300 min^-1^ | 14 mM^-1^* min^-1^ |
| 1-Butanol | 27.5 mM | 235 min^-1^ | 9 mM^-1^* min^-1^ |
| 1-Pentanol | 146.7 mM | 376 min^-1^ | 3 mM^-1^* min^-1^ |
| 1,6-hexanediol | < LOQ^a^ | < LOQ^a^ | < LOQ^a^ |
| Isobutanol | < LOQ^a^ | < LOQ^a^ | < LOQ^a^ |
| Isopropanol | < LOD^b^ | < LOD^b^ | < LOD^b^ |
| Glycerol | < LOD^b^ | < LOD^b^ | < LOD^b^ |
| Glucose | < LOD^b^ | < LOD^b^ | < LOD^b^ |

k_cat_ relates to the turn-over of a single enzyme monomer under substrate saturating conditions. All measurements were conducted at 30°C in 67 mM phosphate-buffer, pH 7.4. Prior to kinetic measurements, the enzyme was determined to have a pH-optimum in the range of pH 7.4 to 8.5.

^a^ LOQ: limit of quantification

^b^ LOD: limit of detection
